# Quality attributes of paddy rice during storage as affected by accumulated temperature

**DOI:** 10.3389/fnut.2023.1337110

**Published:** 2024-01-03

**Authors:** Qian Han, Yifan Chen, Xiuying Liu, Jie Bi, Wei Zhang, Xuefeng Zeng, Pingping Wang, Zaixi Shu

**Affiliations:** ^1^College of Food Science and Engineering, Wuhan Polytechnic University, Wuhan, China; ^2^Key Laboratory for Deep Processing of Major Grain and Oil (Wuhan Polytechnic University), Ministry of Education, Wuhan, China; ^3^School of Liquor and Food Engineering, Guizhou University, Guiyang, China

**Keywords:** rice, storage, accumulated temperature, quality, sensory attributes

## Abstract

In actual storage processes of rice, environment temperatures fluctuate rather than remain constant. Accumulated temperature is the sum of temperature during the storage period. In this research, six different temperature-varied conditions with two accumulated temperatures (low intensity: 7200°C⸱d; high intensity: 9000°C⸱d) were designed to store rice for 12 months and the stored rice samples were compared in quality. Three low-accumulated temperature conditions were set as follows: No. 4–15°C for 6 months followed by 25°C; No. 5–25°C for 6 months followed by 15°C; No. 8-alternating between 15°C and 25°C every 2 months. Similarly, three high-intensity conditions, No. 6, No. 7, and No. 9, were set with a temperature change from 25°C to 35°C. Three constant temperature conditions, No. 1, No. 2, and No. 3, with storage temperature of 15, 25, and 35°C, respectively, were used as controls. Under temperature-varied conditions, rice demonstrated a decline in germination rate (GR), catalase (CAT) and peroxidase (POD) activities, and an increase in fatty acid value (FAV) as storage time increased. After storage, rice exhibited higher water absorption rate (WAR) and volume expansion rate (VER), but reduced stickiness and sensory scores for appearance, taste and overall quality. Generally, three batches at high-accumulated temperature conditions had lower GR and sensory scores, and higher FAV, WAR, and VER compared to those under low-intensity conditions. Furthermore, variations in the sequence of temperature also affected quality parameters, even at the same accumulated temperature. These findings indicate that under temperature-varied conditions, increased accumulated temperature exacerbates rice deterioration, and different temperature sequences can influence quality at a given accumulated temperature.

## 1 Introduction

Rice (*Oryza sativa* L.) is one of the world’s three major staple crops (1, 2). It is cultivated on approximately 150 million hectares of land globally, with a total production of around 440 million tons, accounting for about 30% of the world’s total grain production. The majority of rice production is concentrated in Asia (3, 4). To address natural disasters, conflicts, and the influence of international grain prices on the food market, many nations, including China, have established grain reserve systems to safeguard food security (5).

During the storage period, the quality of rice changes due to metabolic activities. Enzymatic action leads to the hydrolysis and peroxidation of lipids in stored rice, resulting in the production of fatty acids and lipid peroxides, which in turn alter the aroma (6). As a living organism, rice generates harmful reactive oxygen species during storage, which can diminish its physiological vitality (7, 8). Storage also affects the gelatinization properties and cooking qualities of rice (9, 10). Following storage, the starch in rice grains becomes more resistant to gelatinization. Rice cooked from stored grains exhibits increased hardness and decreased palatability compared to fresh rice (11, 12).

The quality deterioration of stored rice is affected by multiple factors, with temperature being a critical factor (13, 14). Increasing storage temperature accelerates the hydrolysis of lipids. In China, the fatty acid value (FAV) is utilized to evaluate the suitability of rice for further storage. Elevated storage temperature suppresses the activity of oxidative enzymes in grains and expedites lipid oxidation (15, 16). Compared to storage at low temperature, storage at high temperature results in more pronounced alterations in rice grain expansion and water absorption capacity during cooking (17–20).

Currently, research on rice storage primarily focuses on constant temperature conditions. However, in actual storage processes, the temperature in the rice storage environment fluctuates rather than remains constant. The effects of temperature-varied conditions on rice quality are still unclear. Recently, accumulated temperature has been utilized to examine the influence of fluctuating storage temperatures on the flavor and microbiota of stored rice (21). In this study, we controlled the temperature at various storage stages to investigate the impact of accumulated temperature on rice quality. This research aims to provide theoretical and technical support for the scientific storage of rice.

## 2 Materials and methods

### 2.1 Reagents and samples

Potassium hydroxide, ethanol, hydrogen peroxide, disodium hydrogen phosphate dodecahydrate, phenolphthalein test solution, and potassium dihydrogen phosphate were purchased from China National Pharmaceutical Group Chemical Reagent Co., Ltd. (Shanghai, China). Potassium permanganate guaiacol was purchased from Tianjin Comio Chemical Reagents Co., Ltd. (Tianjin, China). Concentrated sulfuric acid was purchased from China PingCoal Shenma Group Kaifeng Dongda Chemical Co., Ltd. (Shanghai, China).

The rice samples used in this study were Chinese indica rice (Longliangyou No. 534) harvested in October 2020 from Xiantao City, Hubei Province, China.

### 2.2 Storage conditions of the rice grain samples

The freshly harvested samples were adjusted to a water content below 14.5%, and then packed into a sealed glass container. The samples were stored in a constant temperature incubator (SHX-250B-Z, Boxun, Shanghai, China). The storage conditions were designed with a total of 9 batches, with the first three batches serving as the high-temperature (35°C) control batch, the medium-temperature (25°C) control batch, and the low-temperature (15°C) control batch. Details of the storage conditions were shown in Table 1.

**Table 1:** Storage temperature settings of nine batches.

Batch	Storage temperatures (°C)						Accumulated temperature (°C⸱d)
	1–2 month	3–4 month	5–6 month	7–8 month	9–10 month	11–12 month	
No. 1	15	15	15	15	15	15	5,400
No. 2	25	25	25	25	25	25	9,000
No. 3	35	35	35	35	35	35	12,600
No. 4	15	15	15	25	25	25	7,200
No. 5	25	25	25	15	15	15	7,200
No. 6	15	15	15	35	35	35	9,000
No. 7	35	35	35	15	15	15	9,000
No. 8	15	25	15	25	15	25	7,200
No. 9	15	35	15	35	15	35	9,000

The rice samples were stored for 360 days. During storage, the sample containers were checked regularly to prevent mold formation, and ensure that there was no pest infestation within the containers.

### 2.3 Determination of germination rate and FAV

The indicators of GR and FAV were detected according to the Chinese Standard Methods of GB/T 5520-2011 and GB/T 20569-2006, respectively. The interval for measurements is 60 days.

### 2.4 Evaluation of catalase and peroxidase activity

The CAT activity was detected following the Chinese Standard Method of GB/T 5522-2008.

The POD activity was detected based on Shu et al. (19) method of with some modification. Initially, rice samples were dehusked and ground to a fine powder. In general, 1.0 g of the rice powder was mixed thoroughly with 10 mL of 0.05 mol/L KH_2_PO_4_, followed by centrifugation at 4,000 r/min for 10 min. The resultant supernatant was used as the enzyme extract.

For the reaction mixture, 56 μL of guaiacol was introduced into 100 mL of phosphate buffer (1 mol/L, pH 6.0) and stirred at 40°C for 20 min. Upon cooling, 38 μL of 30% hydrogen peroxide solution was added. In a cuvette, 1 mL of the enzyme extract was combined with 3 mL of the reaction mixture, and the reaction was allowed to proceed for 3 min. The absorbance of the reaction mixture was measured at 470 nm using a UV–visible spectrophotometer (T6, Persee, Beijing, China). POD activity was calculated according to the Equation (1),


(1) $$\mathrm{POD}\;\mathrm{activity}/U=\frac{\Delta D_{470}*V_{T}}{W*V_{S}*0.01}\left[U/\left(g\cdot \min\right)\right]$$

where ΔD_470_ was the change of absorbance before and after the reaction, *w* was the mass of the samples (g), V_T_ was the total volume of the sample solution (mL), and *V_S_* was the volume of the enzyme solution for detection (mL).

### 2.5 Transmitting electron microscope

Midsection samples, with a thickness ranging from 0.25 to 0.5 mm, were extracted from the germ of rice and subjected to fixation in a 4% glutaraldehyde solution (v/v). Following fixation, the samples were rinsed four times, each for 15 min, using a 0.2 mol/L phosphate buffer saline (PBS, pH 7.2). The samples were then post-fixed in 1% osmic acid (w/v) for 90 min. Subsequent to post-fixation, the samples were rinsed thrice, each for 15 min, with 0.2 mol/L pH 7.2 PBS solution, and dehydrated in an increasing series of acetone solutions (30, 50, 70, 90, and 100% v/v), each for 15 min. The samples were then embedded in an EPON 812 resin/acetone series (1:2, 1:1, 2:1 and 1:0), each for 120 min. The embedded samples were oven-dried at temperatures of 37°C, 45°C, and 60°C, each for 12 h. The plastic molds were trimmed and a section of 80 nm was prepared using a rotary microtome. The section was stained first with uranyl acetate for 25 min and then with lead citrate for 20 min. The stained samples were observed and photographed using a JEM-1200EX TEM (Jeol, Japan).

### 2.6 Analysis of cooking characteristics

The samples were dehusked, and then were milled using an automatic rice mill. For sample preparation, 50 mL distilled water was added to 7 ± 0.01 g of milled rice (m_1_), and the total volume was recorded as V_1_. Then, the rice was washed with distilled water twice, and placed in a mesh bag, boiled with 120 mL water for 20 min. After boiling, the remaining rice was taken out from the cooking liquid. The pH value of cooking liquid was measured with a pH Meter (FE28, Mettler Toledo, Shanghai, China). The mass of the prepared rice was weighed and recorded as m_2_. 50 mL distilled water was added to the prepared rice, and the total volume was recorded as V_2_.

Water absorption ratio (WAR) and volume expansion ratio (VER) was calculated using the Equation (2) and Equation (3), respectively.


(2) $$\mathrm{WAR}=\frac{m_{2}}{m_{1}}$$

(3) $$VER=\frac{V_{1}-50}{V_{2}-50}$$

### 2.7 Evaluation of texture and sensory quality of cooked rice

The milled rice was washed thoroughly in distilled water, and then was cooked in an automatic rice cooker (MG-TH559, Median, Foshan, China) for 30 min. The ratio of rice to water was 1:1.5 (m/m). The cooked rice was molded into rice balls, and then was placed in a Rice Taste Meter (STA1B, Satake, Japan) to determine the rice sensory indicators including appearance score, and taste score, and the overall score. Hardness, stickiness, and springiness were determined using and a Hardness Viscometer (RHS1A, Satake, Japan).

### 2.8 Data analysis

Statistical analysis was performed using Duncan’s multiple range test to determine significant differences between means. The data were expressed as mean ± standard deviation (SD). All statistical analyses were conducted using the SPSS software package (SPSS, IL, United States).

## 3 Results and discussion

### 3.1 GR

**Figure 1:**
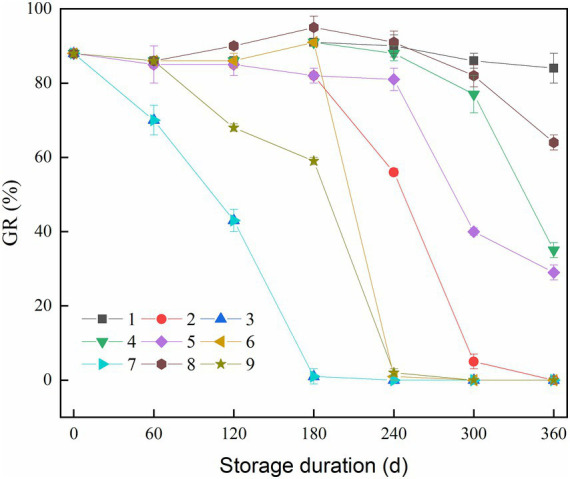
Change in germination rate (GR) of rice during storage.

The GR of rice samples is a key indicator for assessing the freshness, growth potential, and vitality of rice kernels (22, 23). As shown in Figure 1, an overall decreasing trend in the GR of the rice samples was observed for 9 batches. Notably, for control batches (No. 1, No. 2, and No. 3), as the storage temperature increased, the decline speed in GR became faster. The GR reached 0% after 180 days of storage at a temperature of 35°C, and after 300 days of storage at a temperature of 25°C, respectively. This indicates that the samples would lose their germination ability under medium to high temperature conditions. However, at a lower storage temperature of 15°C, the GR only fell by about 20% during the entire storage period.

The GR of batches No. 4, No. 5, and No. 8 declined progressively over time. Among them, the results of batch No.8 were better than the other two groups. This result suggested that temperature variations at different stages can influence the viability of rice kernels, even under the same accumulated temperature conditions. Batches No. 4 and No. 5 had a storage period exceeding 180 days under the temperature setting of 25°C, resulting in a slightly faster falling rate.

For the batches with a high temperature storage period, the GR of batches No. 6, No. 7, and No. 9 demonstrated a marked decrease during the period at 35°C, eventually dropping to 0%. Notably, batch No. 7, initially stored at 35°C, did not recover GR even after the temperature was subsequently lowered to 15°C. This is likely due to the fact that the rice grains completely lost their germination ability at the initial high-temperature stage (24). The comparison of batches with different accumulated temperatures (No. 4, No. 5, and No. 8: 7200°C⸱d; No. 6, No. 7, and No. 9: 9000°C⸱d) revealed that a higher accumulated temperature exacerbated the viability reduction in rice kernels.

### 3.2 FAV

**Figure 2:**
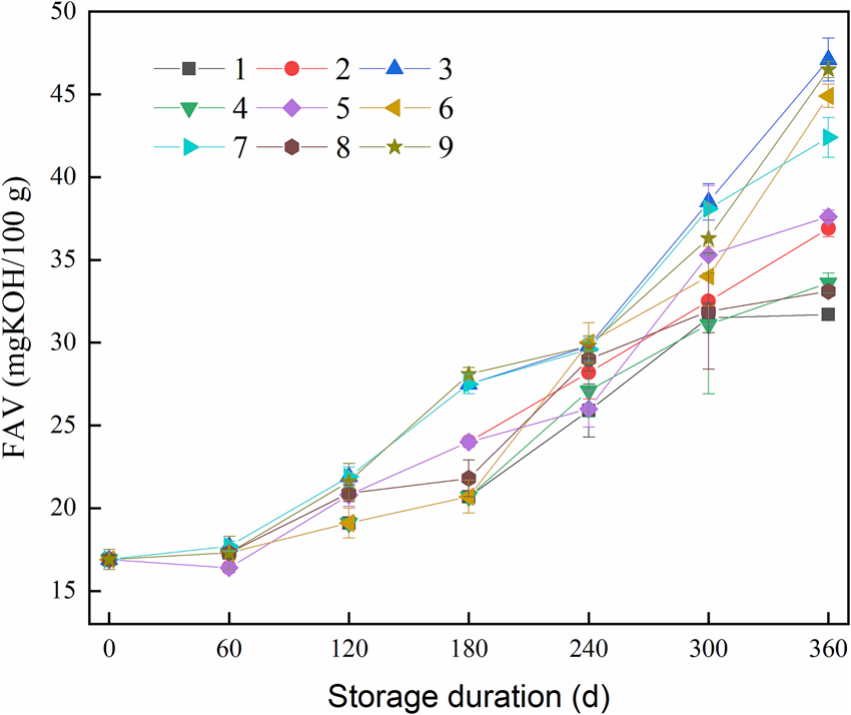
Change in fatty acid value (FAV) of rice during storage.

The FAV serves as an indicator of rice quality and nutritional value (25, 26). Generally, FAV increases during storage, as evidenced in Figure 2. FAV increased approximately 2-fold under constant high-temperature conditions of 35°C (batch No. 3) compared to low-temperature conditions of 15°C (batch No. 1). The final FAVs of batches No. 4, No. 5, and No. 8 were 33.6, 37.6, and 33.1 mg KOH/100 g, respectively, while the value of the batches No. 6, No. 7, and No. 9 were 44.9, 42.4, and 46.5 mg KOH/100 g, respectively. These results suggested that a rise in accumulated temperature accelerated fat oxidation in rice, and at the same accumulated temperature, the variation of temperature history can make a difference in aging process.

### 3.3 POD and CAT activities

**Figure 3:**
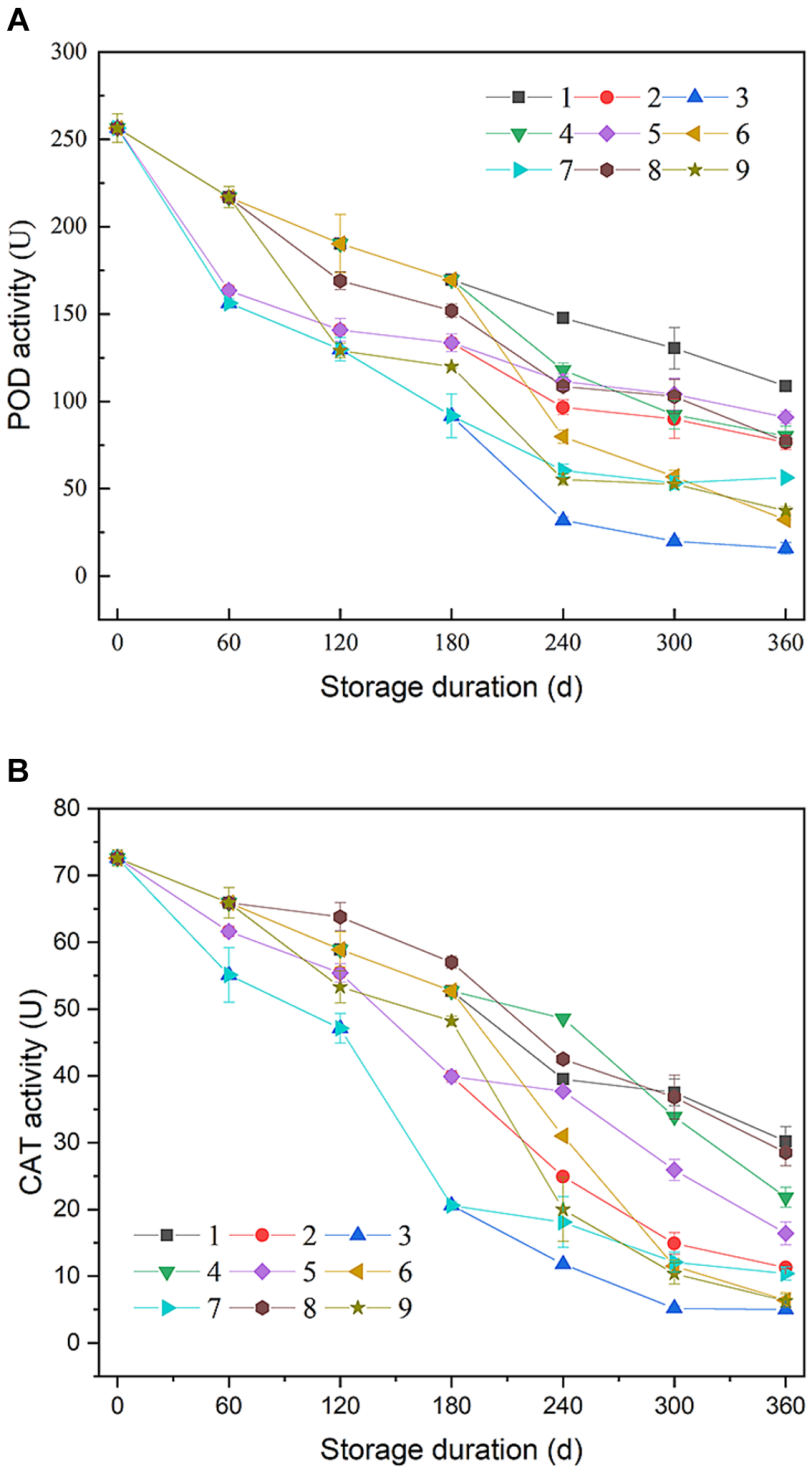
Change in peroxidase (POD) and catalase (CAT) activities of rice during storage. **(A)** POD; **(B)** CAT.

As shown in Figure 3A, during the storage, rice showed a downward trend in POD activity. This result was consistent with Zhu et al. (10). The peroxidase activity can be used as an indicator for freshness of rice (27). It was observed that different storage conditions led to varying rates of enzyme activity decline. In control batches No. 1, No. 2, and No. 3, the decline rate of POD activity in batch No.3 (samples storage at 35°C) was approximately twice as fast as in batch No. 1 (samples storage at 15°C). For the batches No. 5, and No. 7, POD activity decline was quicker between 60 and 180 days and then slowed from 180 to 360 days. This patter corelated with the initial high temperature followed by cooler storage periods. Conversely, batches No. 4, and No. 6 exhibited a slow decline that accelerated later. This could be attributed to the temperature increase during storage. Batch No. 6, in particular, showed a rapid late-stage decline due to a temperature rice to 35°C. Batches No. 4, and No. 6 showed fluctuating decline trend, and the fluctuation could be due to alternating temperature changes during storage. The trends in CAT activity changes across different batches were similar to those of POD activity (Figure 3B).

After the entire storage process, the final POD and CAT activities of batches No. 4, No. 5, and No. 8 intermediate between batch No. 1 and No. 2, while activities of the two enzymes in batches No. 6, No. 7, and No. 9 were between batch No. 2 and No. 3. These results implied that higher accumulated temperature can intensify the inactivation of POD and CAT. There was also differences in enzyme activities within batch groups at the same accumulated temperature, suggesting that temperature order variation can affect POD and CAT activities.

### 3.4 Microstructure analysis of rice during storage

**Figure 4:**
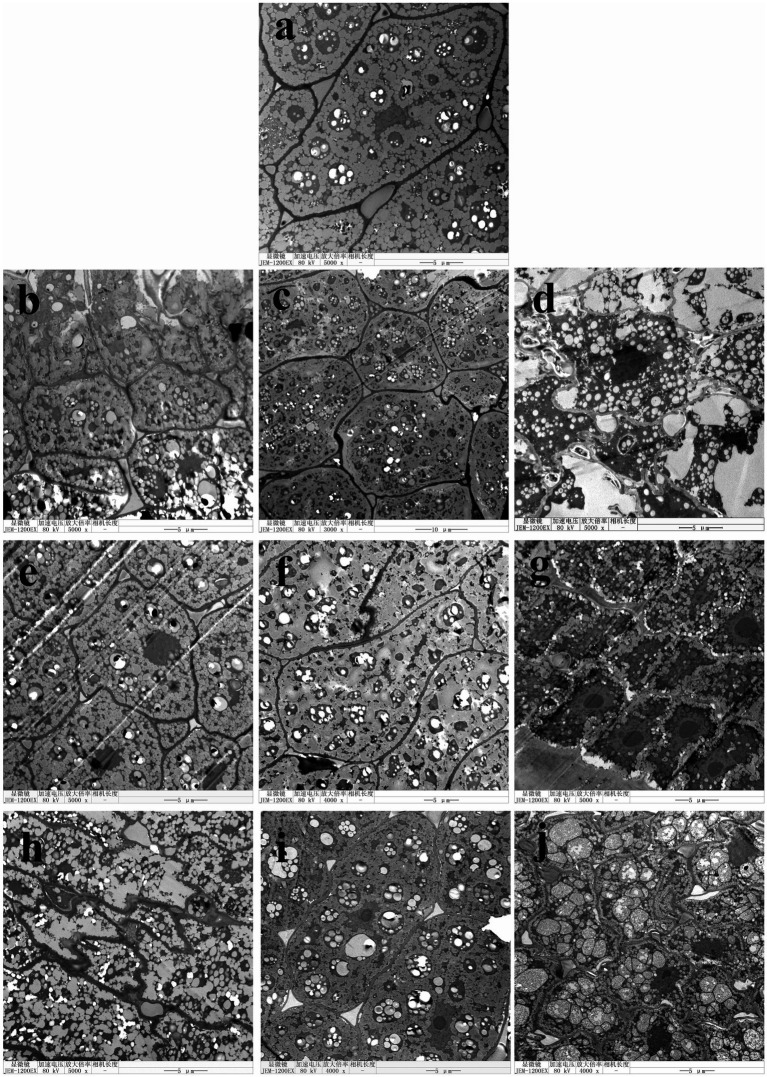
Microstructure images of germs of rice after storage. **(A)** fresh rice; **(B)** batch No. 1; **(C)** batch No. 2; **(D)** batch No. 3; **(E)** batch No. 4; **(F)** batch No. 5; **(G)** batch No. 6; **(H)** batch No. 7; **(I)** batch No. 8; **(J)** batch No. 9.

As shown in Figure 4, the germ cell structure of the original rice samples was intact, with clear intercellular spacing and dense, undamaged cell walls. The cell membrane was closely adhered to the cell walls, and organelles were evenly distributed. After 360 days of storage, batches No. 1, No. 2, No. 4, No. 5, No. 8 maintained the clear cell wall and membrane structures. However, batches No. 3, No. 6, No. 7, and No. 9 displayed significant cellular structure alterations; some organelles appeared fluid-like, and the shape of protein storage vacuoles was distorted. These results suggested that the increased accumulated temperature could result in damage to the cellular structure of rice germ. At temperature-varied condition, batches with same accumulated temperature still had slight difference in the structure of germ cell, indicating that the change of temperature order and fluctuation frequency can lead to differences in the extent of high temperature-induced impairment.

### 3.5 Cooking characteristics of rice

As Table 2 shows, comparing to the freshly prepared samples, the storage process led to varying extent of pH reduction in the cooking liquid for all 9 batches. The decrease of pH can be attributed to the accumulation of acidic by-products from fat rancidity (28). For three control batches, pH values of cooking liquid of batches No. 1 and No. 2 were higher than that of batch No. 3. This result was generally in agreement with the results of FAV. Batches No. 4 to No. 9 exhibited only minor differences in pH values of cooking liquid.

**Table 2:** Changes of cooking quality after 360 days of storage*.

Batch	pH of cooking liquid	WAR (%)	VER (%)
Fresh rice	6.8 ± 0.1^e^	311 ± 14^a^	389 ± 31^ab^
No. 1	6.5 ± 0.2^d^	314 ± 12^a^	384 ± 12^a^
No. 2	6.4 ± 0.1^cd^	338 ± 13^bc^	410 ± 36^abc^
No. 3	5.9 ± 0.0^a^	318 ± 15^a^	388 ± 26^ab^
No. 4	6.1 ± 0.1^ab^	323 ± 9^ab^	394 ± 34^ab^
No. 5	6.3 ± 0.0^bcd^	327 ± 1^abc^	403 ± 12^abc^
No. 6	6.3 ± 0.0^bcd^	384 ± 10^d^	480 ± 23^d^
No. 7	6.2 ± 0.1^bc^	346 ± 11^c^	435 ± 25^bcd^
No. 8	6.4 ± 0.1^cd^	319 ± 1^a^	400 ± 24^abc^
No. 9	6.2 ± 0.0^bc^	345 ± 4^c^	443 ± 23^cd^

*Data were expressed as means ± SD. Means within a column that had the same letter were not significantly different (α = 0.05). WAR, water absorption rate; VER, volume expansion rate.

After rice storage, batches No. 1 and No. 3 showed no significant change in WAR and VER of cooked rice, while batch No. 2 experienced a notable decrease in these cooking quality parameters. This result was consistent with Zhou et al. (29) and Swamy et al. (30). For fresh rice and rice stored at low temperature, the moisture held in the cooked rice is mainly involved in the starch hydration. As aging processes, the rice kernels’ resistance to hydrothermal disruption increases, and moisture becomes partially involved in starch gelatinization, with some trapped due to volume expansion. However, further aging reduces hydration capacity, leading to lower water absorption and volume expansion. At temperature-varied conditions, batches with accumulated temperature of 7,200°C⸱d generally had lower WAR and VER than those at 9,000°C⸱d, indicating that higher accumulated temperature can, to some extent, increase water absorption and volume expansion. There were no significant different in WAR and VER among batches No. 4, No. 5, and No. 8, but batches No. 6, No. 7, and No. 9 exhibited variations in these parameters, suggesting that temperature sequence changes can influence the cooking quality of rice.

### 3.6 Texture and sensory attributes of cooked rice

Rice texture and sensory attributes, including hardness, stickiness, appearance, taste, and overall score, testing with a taste meter and hardness viscometer. As shown in Table 3, after storage, rice displayed a significant decrease in stickiness and springiness of cooked rice. The lower stickiness and springiness are likely associated with the reduced starch granule hydration from aging. However, no differences were found among stored rice samples in these texture parameters. Stored rice generally scored lower in appearance, taste, and overall quality compared to fresh rice. At temperature-varied conditions, rice stored at low accumulated temperature (7,200°C⸱d) generally had higher sensory scores than those stored at higher accumulated temperature (9,000°C⸱d). These results suggested that elevation of accumulated temperature would decrease the sensory acceptance of rice. Nonetheless, no significant sensory score differences were noted among batches with the same accumulated temperature.

**Table 3:** Changes of taste quality after 360 days of storage*.

Batch	Hardness	Stickiness	Springiness	Appearance score	Taste score	Overall score
Fresh rice	0.857 ± 0.263^a^	0.105 ± 0.007^c^	0.887 ± 0.006^b^	5.4 ± 1.3^c^	5.3 ± 1.6^b^	65.3 ± 10.2^c^
No. 1	0.685 ± 0.007^a^	0.050 ± 0.000^b^	0.780 ± 0.014^a^	4.2 ± 0.1^abc^	4.2 ± 0.1^ab^	55.1 ± 1.8^abc^
No. 2	0.660 ± 0.085^a^	0.040 ± 0.014^ab^	0.815 ± 0.049^a^	3.9 ± 1.0^abc^	4.0 ± 1.1^ab^	53.0 ± 7.4^abc^
No. 3	0.735 ± 0.219^a^	0.020 ± 0.000^a^	0.810 ± 0.081^a^	3.6 ± 0.5^ab^	4.0 ± 0.5^ab^	53.8 ± 3.3^abc^
No. 4	0.580 ± 0.014^a^	0.040 ± 0.000^ab^	0.810 ± 0.042^a^	5.0 ± 0.1^bc^	5.1 ± 0.2^b^	61.2 ± 0.8^bc^
No. 5	0.525 ± 0.035^a^	0.030 ± 0.000^ab^	0.800 ± 0.028^a^	4.2 ± 0.4^abc^	4.2 ± 0.4^ab^	54.0 ± 2.5^abc^
No. 6	0.695 ± 0.078^a^	0.030 ± 0.000^ab^	0.805 ± 0.007^a^	3.0 ± 0.2^a^	3.1 ± 0.2^a^	47.3 ± 2.3^a^
No. 7	0.645 ± 0.021^a^	0.025 ± 0.000^ab^	0.825 ± 0.007^a^	3.5 ± 0.4^ab^	3.5 ± 0.4^ab^	50.0 ± 2.3^ab^
No. 8	0.580 ± 0.085^a^	0.040 ± 0.014^ab^	0.810 ± 0.000^a^	4.0 ± 0.3^abc^	4.4 ± 0.2^ab^	54.2 ± 1.6^abc^
No. 9	0.600 ± 0.198^a^	0.030 ± 0.000^ab^	0.810 ± 0.014^a^	3.3 ± 0.2^a^	3.7 ± 0.4^ab^	50.0 ± 2.6^ab^

*Data were expressed as means ± SD. Means within a column that had the same letter were not significantly different (α = 0.05).

## 4 Conclusion

Over time, rice stored under varied temperature conditions exhibited declining GR, POD, and CAT activities, and increasing FAV. After storage, the change in these parameters were more pronounced at higher accumulated temperature, which also corresponded to higher WAR and VER and lower sensory scores. Differences in rice quality attribute were also observed among batches at the same accumulated temperature, indicating that temperature history can influence rice quality. These findings offer theoretical and practical insights for paddy rice storage. Further research work will be more focused on the physiological and biochemical metabolism of rice at different stages of temperature-varied conditions.

## Data availability statement

The original contributions presented in the study are included in the article/supplementary material, further inquiries can be directed to the corresponding authors.

## Author contributions

QH: Investigation, Writing – original draft. YC: Investigation, Writing – original draft. XL: Validation, Writing – review & editing. JB: Writing – original draft. WZ: Funding acquisition, Visualization, Writing – review & editing. XZ: Validation, Writing – review & editing. PW: Conceptualization, Methodology, Writing – original draft. ZS: Conceptualization, Visualization, Writing – original draft.
